# Supplementary figures and images for: The intrinsic chaperone network of Arabidopsis stem cells confers protection against proteotoxic stress
Source: Aging Cell. 2021 Jul 30;20(8):e13446. doi: 10.1111/acel.13446 (PMC8373342; doi:10.1111/acel.13446)

Hoechst

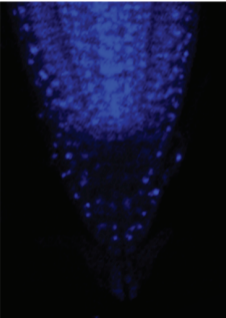

ProteoStat

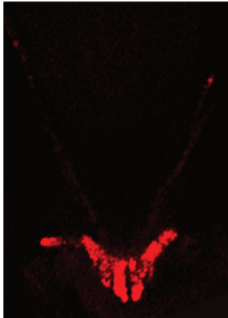

Bright field

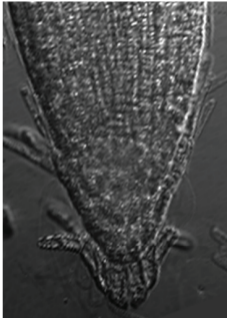

Merge

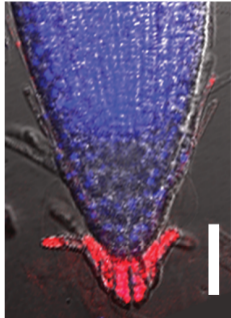

Supplement: Supplementary file 2 — Fig S1 [file ACEL-20-e13446-s006.pdf]

**a**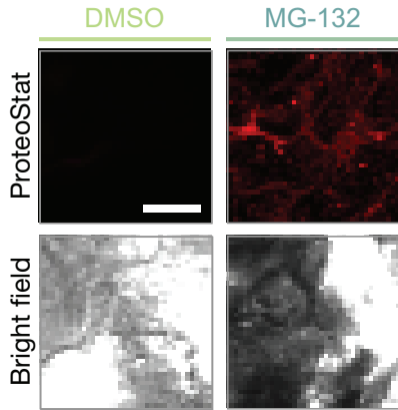**b**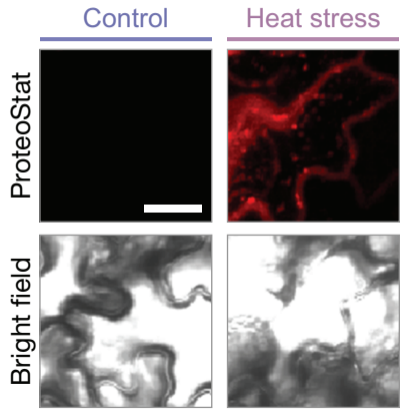

Supplement: Supplementary file 3 — Fig S2 [file ACEL-20-e13446-s014.pdf]

**a**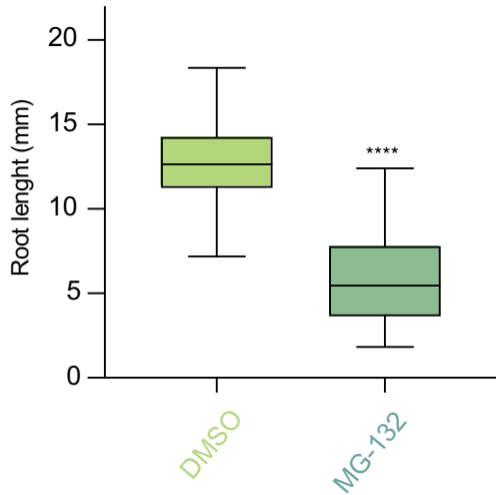**b**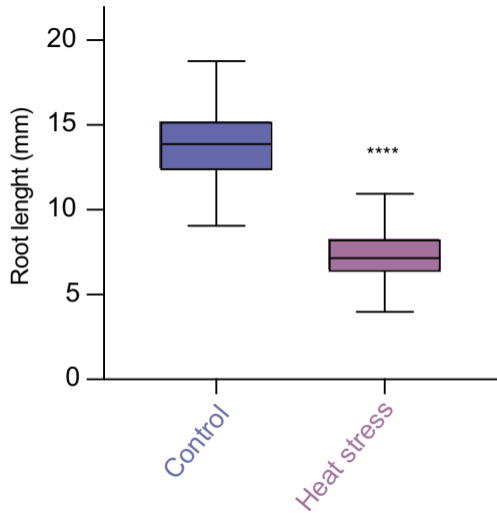

Supplement: Supplementary file 4 — Fig S3 [file ACEL-20-e13446-s001.pdf]

**a**

Control 22 °C

37°C heat stress (HS)  
for 2 days at 4 DAG,  
recover for 5 days at  
22°C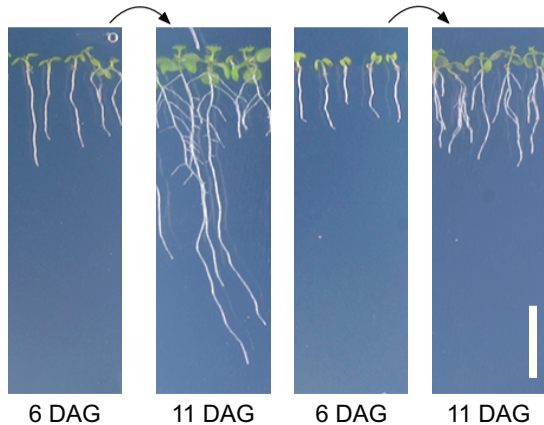**b**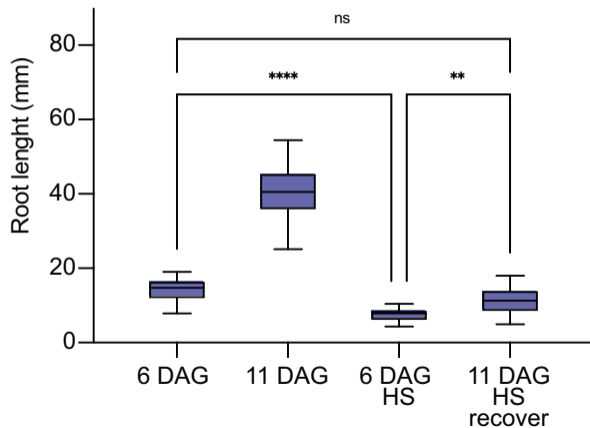

Supplement: Supplementary file 5 — Fig S4 [file ACEL-20-e13446-s005.pdf]

**a**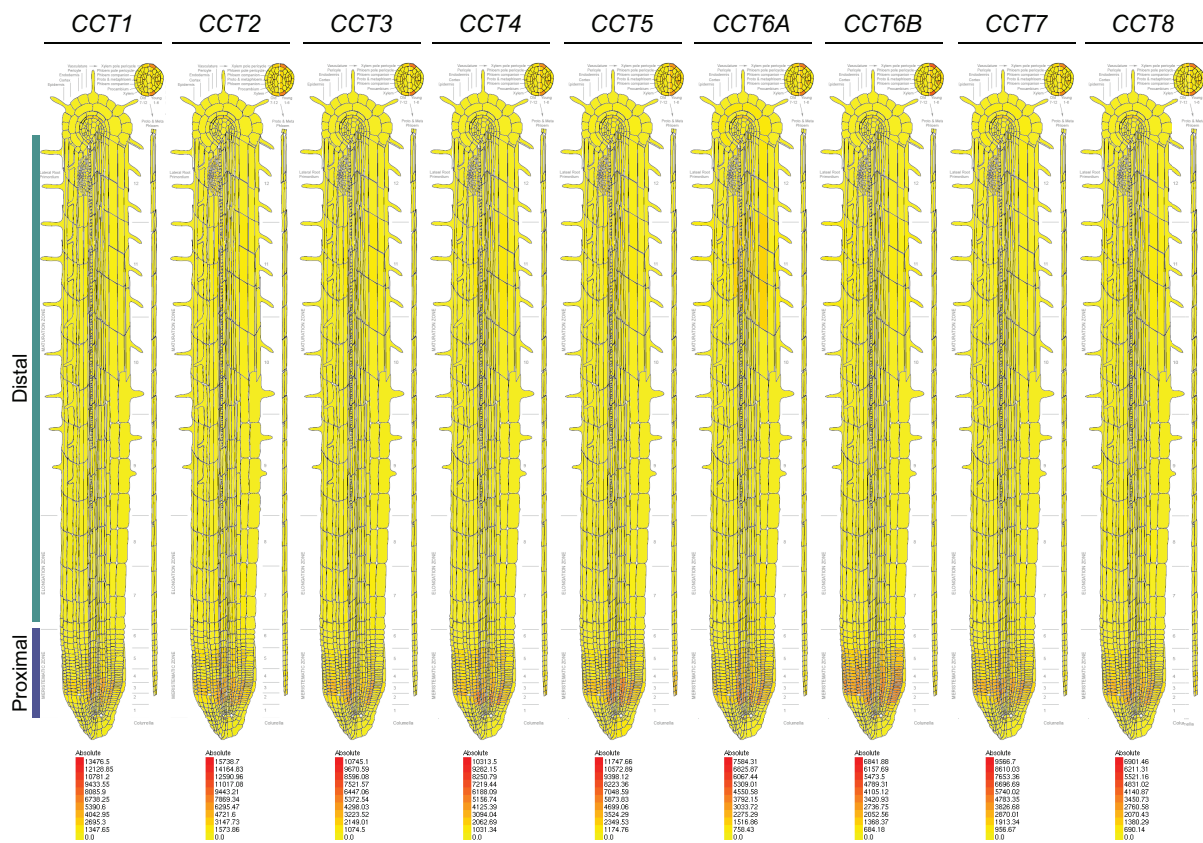**b**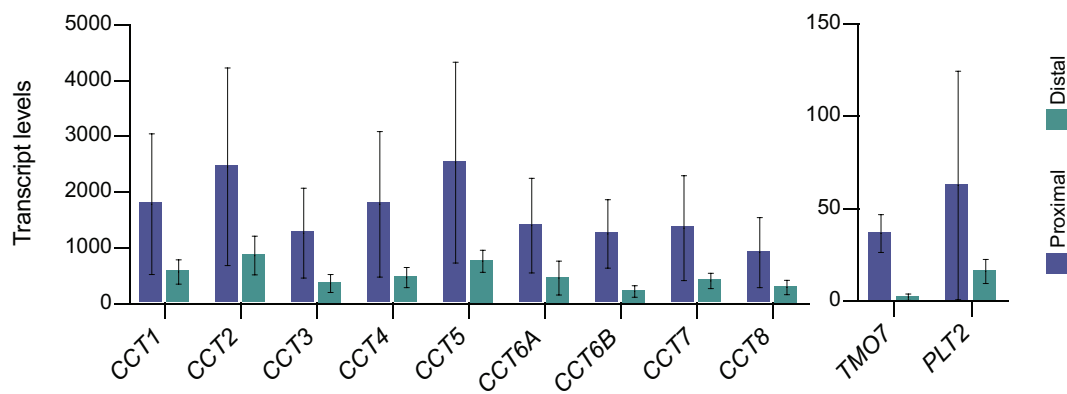

Supplement: Supplementary file 6 — Fig S5 [file ACEL-20-e13446-s009.pdf]

**a**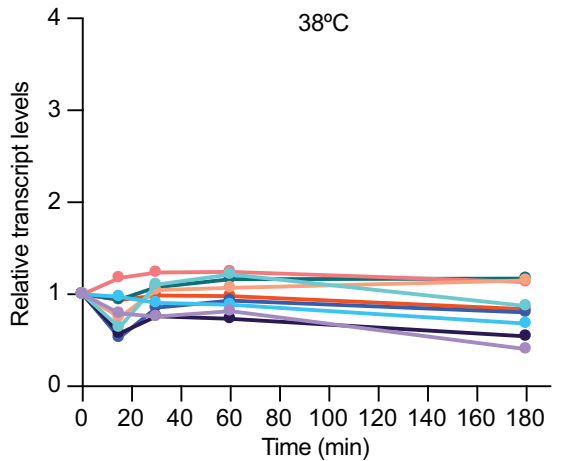

— CCT1      — CCT4      — CCT6B  
— CCT2      — CCT5      — CCT7  
— CCT3      — CCT6A      — CCT8

**b**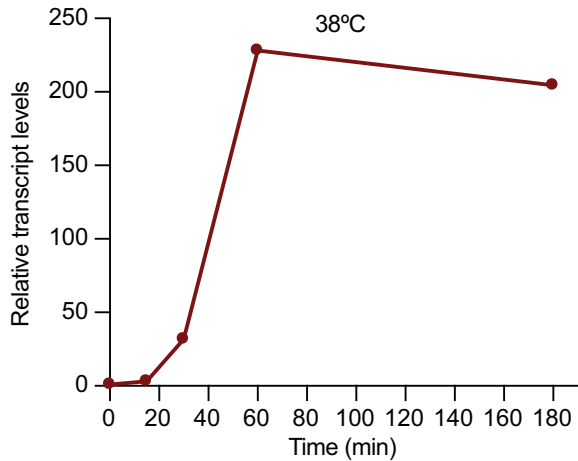

— HsfA2

Supplement: Supplementary file 7 — Fig S6 [file ACEL-20-e13446-s002.pdf]

**a**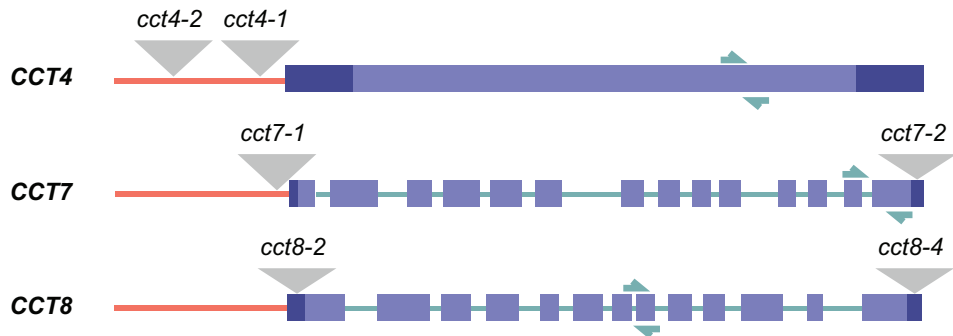**b**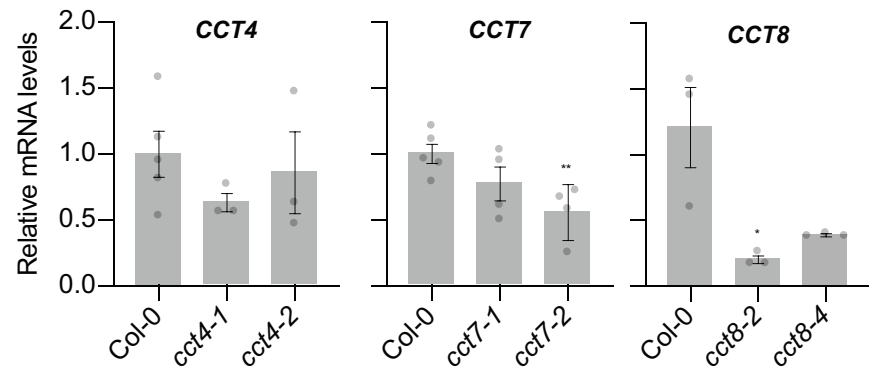**c**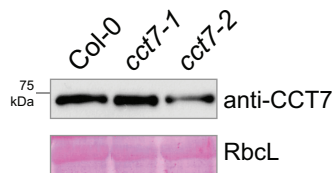

Supplement: Supplementary file 8 — Fig S7 [file ACEL-20-e13446-s012.pdf]

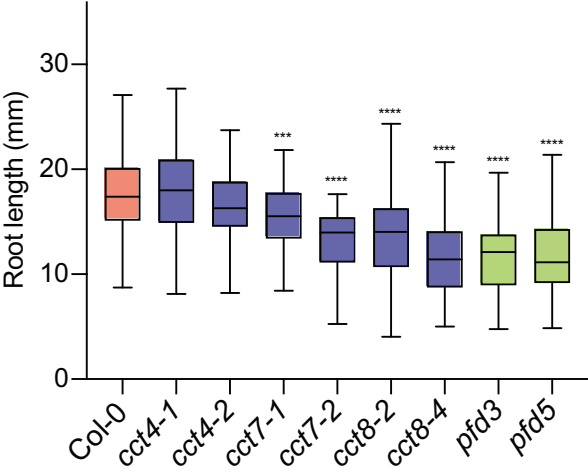

Supplement: Supplementary file 9 — Fig S8 [file ACEL-20-e13446-s008.pdf]

**a**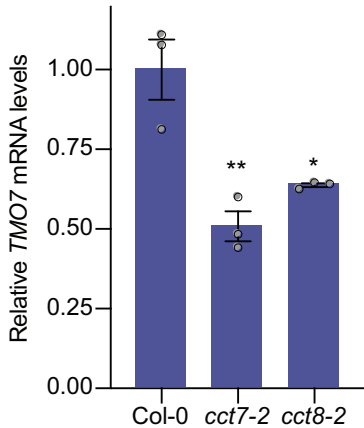**b**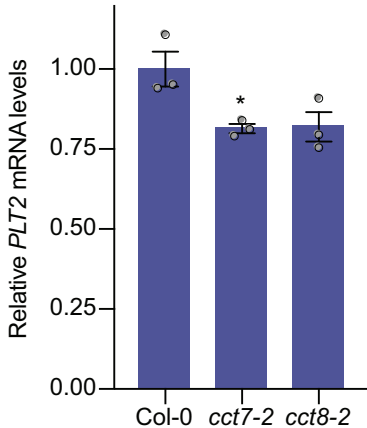

Supplement: Supplementary file 10 — Fig S9 [file ACEL-20-e13446-s004.pdf]

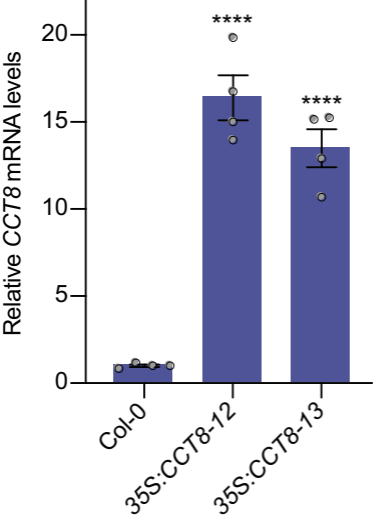

Supplement: Supplementary file 11 — Fig S10 [file ACEL-20-e13446-s007.pdf]

**a**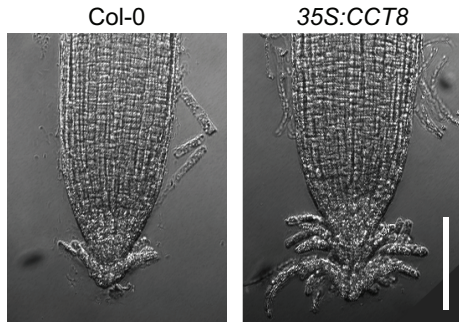**b**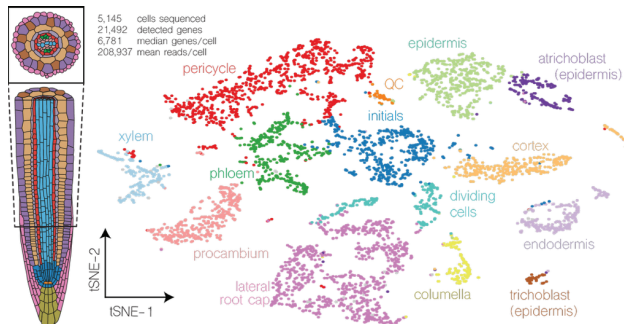**c**

CCT8 expression pattern in columella

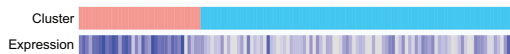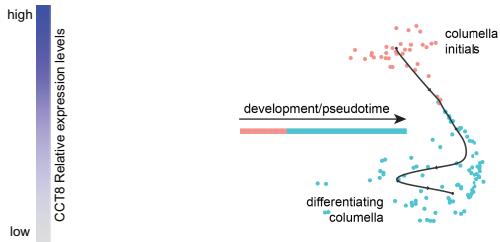

CCT8 expression pattern in lateral root cap

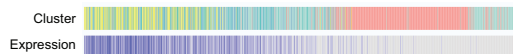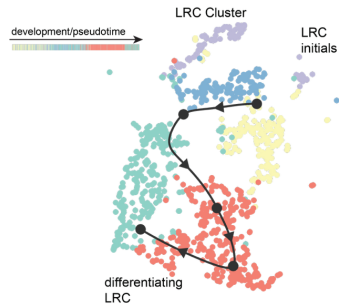

Supplement: Supplementary file 12 — Fig S11 [file ACEL-20-e13446-s010.pdf]

**a**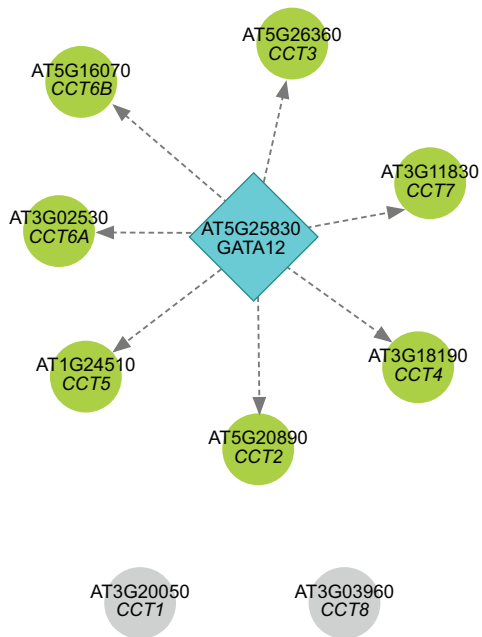**b**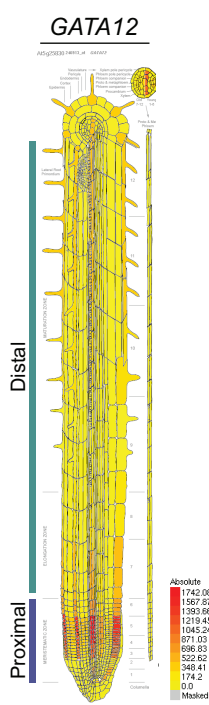**c**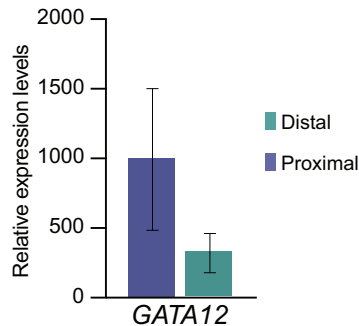

Supplement: Supplementary file 13 — Fig S12 [file ACEL-20-e13446-s015.pdf]
