# Supplementary material for: The intrinsic chaperone network of Arabidopsis stem cells confers protection against proteotoxic stress
Source: Aging Cell. 2021 Jul 30;20(8):e13446. doi: 10.1111/acel.13446 (PMC8373342; doi:10.1111/acel.13446)
Supplement: Supplementary file 14 — Tab S1 [file ACEL-20-e13446-s011.pdf]

Gene	AGI Locus	Primers used for qPCR (F/R)	Reference
CCT1	AT3G20050	GTC GGC CTC GAC AAG ATG<br>CGA CCT CCA ACA TCC TAA GAA	Ahn et al., 2019. J Exp Bot. 70: 2741-2757
CCT2	AT5G20890	CTA AGA TTC ACC CTA TGA CCA TCA<br>TTC AGT AAA GCA TTA CGA GCA CA	Ahn et al., 2019. J Exp Bot. 70: 2741-2757
CCT3	AT5G26360	CGC TCA TCC TGC AGC TAA GT<br>ACC AGC TAG AAC AAT AAC AGA CGT T	Ahn et al., 2019. J Exp Bot. 70: 2741-2757
CCT4	AT3G18190	AGT GAG CAA AAG GTT TTT GAT TG<br>ACC GAG CTG CCT TGA GAG	Ahn et al., 2019. J Exp Bot. 70: 2741-2757
CCT5	AT1G24510	TTG CTC ACT TGC GGT TGA T<br>GCA AAC GCC CTA ATT GCA TA	Ahn et al., 2019. J Exp Bot. 70: 2741-2757
CCT6A	AT3G02530	AGT GCC TAA GAC GCT TGC TG<br>TCC TTT GTC ATG CTC ACT CG	Ahn et al., 2019. J Exp Bot. 70: 2741-2757
CCT6B	AT5G16070	GAA AGC CTG AGG AAG CTA TTG A<br>GAA CAA GCC CCT CAA CCA	Ahn et al., 2019. J Exp Bot. 70: 2741-2757
CCT7	AT3G11830	CGACTGAAGCGGCTTGCCTCAT<br>TATCGCCTTCGCATTCCACGGC	This study
CCT8	AT3G03960	GTC GCC CAT TTG AAG CTT AG<br>CTC GTT TCT TGC AAT CGT GA	Ahn et al., 2019. J Exp Bot. 70: 2741-2757
TMO7	AT1G74500	CGGGAAGAAGATCACGTTCGAGGC<br>GGAACGACGACTGTCCCTGAGC	This study
PLT2	AT1G51190	GGTAGGGTATGGAATAATTAGC<br>CCTAAAAAGACTAACCCTCGAG	Durgaprasad et al., 2019. Cell Rep. 29: 453-463
EF1α	AT5G60390	CTGGAGGTTTTGAGGCTGGTAT<br>CCAAGGGTGAAAGCAAGAAGA	Wendrich et al., 2017. PNAS. 114: E8922-E8929
PP2A	AT1G13320	TAACGTGGCCAAAATGATGC<br>GTTCTCCACAACCGCTTGGT	Roth et al., 2018. Plant Cell. 30: 1309-1321
